# Integration of Artificial Intelligence and Wearable Devices in Pediatric Clinical Care: A Review

**DOI:** 10.3390/bioengineering12121320

**Published:** 2025-12-03

**Authors:** Huili Zheng, Pragya Sharma, Matthew Johnson, Matteo Danieletto, Eugenia Alleva, Alexander W. Charney, Girish N. Nadkarni, Chethan Sarabu, Bjoern M. Eskofier, Yuri Ahuja, Florian Richter, Eyal Klang, Sandeep Gangadharan, Felix Richter, Emma Holmes, Benjamin S. Glicksberg

**Affiliations:** 1 Windreich Department of Artificial Intelligence and Human Health, Icahn School of Medicine at Mount Sinai, New York, NY 10029, USA; huili.zheng@mssm.edu (H.Z.); pragya.sharma@mssm.edu (P.S.);; 2 The Hasso Plattner Institute for Digital Health at Mount Sinai, Icahn School of Medicine at Mount Sinai, New York, NY 10029, USA; 3 The Center for AI in Children’s Health, Icahn School of Medicine at Mount Sinai, New York, NY 10029, USA; 4 Health Tech Hub, Cornell Tech, Weill Cornell Medicine, New York, NY 10044, USA; 5 Institute of AI in Medicine, LMU Munich & LMU Hospital, 85152 Munich, Germany; 6 Institute of AI for Health, Helmholtz Munich, 85764 Neuherberg, Germany; 7 ArtemisAI Labs, Inc., San Francisco, CA 94102, USA; 8 Department of Pediatrics, Kravis Children’s Hospital, New York, NY 10029, USA; 9 Children’s Hospital of Philadelphia, Philadelphia, PA 19104, USA

**Keywords:** pediatrics, wearable devices, artificial intelligence, wearables, biosignals, healthcare

## Abstract

Wearable devices are becoming widely applied in healthcare to enable continuous, noninvasive monitoring, but their use in pediatric populations remains relatively underexplored. This review synthesizes 36 clinical studies focused on pediatric hospital and outpatient wearables published between 2014 and 2025. Devices included wrist-worn trackers, adhesive biosensors, and more, capturing electrocardiography, photoplethysmography, accelerometry, and other signals. Clinical applications spanned a variety of care settings. Artificial intelligence (AI) partially enhanced interpretation for the early detection of conditions such as postoperative complications and sepsis. Despite their promising accuracy, most studies remain small, single-center pilots focused on feasibility and signal validity rather than outcomes such as mortality, readmission, or long-term recovery. Key barriers include pediatric-specific device design, motion-robust signal quality, regulatory clearance, workflow integration, and equitable adoption in low-resource settings. Ethical concerns such as privacy, consent, and incidental findings and regulatory constraints, particularly the lack of pediatric labeling and approval for consumer and AI-driven devices, further limit translation into practice. Future work should prioritize multi-center studies, multimodal analytics, explainable AI, and seamless integration into clinical pathways. With these advances, wearables can move beyond feasibility to become reliable, personalized tools that improve pediatric monitoring and care.

## 1. Introduction

Wearable devices (WDs) have transformed how continuous biosignals can be captured, interpreted, and linked to clinical action. Consumer technologies such as Fitbit and Apple Watch have normalized the idea of passive tracking, while medical-grade patches and textile sensors now allow for the high-resolution acquisition of electrocardiography (ECG), photoplethysmography (PPG), accelerometry, electrodermal activity (EDA), temperature, and acoustic data. In adults, WDs are widely studied for arrhythmia detection, sleep monitoring, and activity tracking [1,2,3]. Pediatric applications require targeted examinations, as children are not simply “small adults”: they exhibit higher heart and respiratory rates, developmental changes, and diverse disease profiles which manifest in different forms as they age [4]. They may require parental or staff assistance with device application, charging, and troubleshooting. Devices must therefore be designed to be comfortable, safe, and tolerable for fragile skin and smaller body sizes, and should accommodate the accessibility requirements of the patients they are targeted towards. Furthermore, they need to be validated specifically in pediatric populations.

The clinical case for pediatric WDs is strong. At present, patients are routinely kept for monitoring after operations, but WDs could enable earlier home monitoring and thus enable earlier discharge [5]. Wireless continuous monitoring could support environments such as emergency departments that are facing rising patient volumes by monitoring patients for adverse events while they are waiting [6]. Beyond acute care, WDs may offer new approaches to chronic disease management, rehabilitation, and even preventive pediatrics [7,8,9]. Another key opportunity, particularly in very young children and infants, lies in the fact that these patients have limited ability to communicate subjective health states. Objective, passively collected physiological data from WDs can therefore provide a valuable alternative means to assess disease progression or recovery after hospital discharge.

AI adds another layer: by analyzing noisy, high-frequency biosignal data, often in conjunction with other clinical data streams, machine learning can uncover patterns that are invisible to clinicians or too complex for traditional algorithms [10]. The convergence of sensors and AI raises the prospect of moving from monitoring to proactive intervention in pediatric care, as well as the more quantitative tracking of developmental milestones. 

Despite enthusiasm, the literature is fragmented. Most reports are single-center feasibility studies, and many focus on validating signals against gold-standard monitors rather than demonstrating improved outcomes. Hard clinical endpoints such as mortality, readmissions, disease-specific endpoints, and long-term quality of life are rarely assessed. AI applications are promising but are limited by small sample sizes, heterogeneous data, and a lack of external validation. 

This review aims to synthesize current evidence on pediatric hospital and outpatient wearables, with particular attention to AI-driven applications. We catalog the types of devices used, examine their clinical applications across specialties, evaluate how AI has been integrated, and analyze the challenges of implementation.

Recent scoping and systematic reviews have begun mapping the landscape of pediatric biosensors, particularly in inpatient settings. For example, Hyun et al. (2025) [11] identified more than 100 wearable sensors used for hospitalized children and highlighted gaps in clinical validation, reliability testing, and complication reporting. Similarly, Grooby and Sitaula et al. (2023; Part 1 and Part 2) [12,13] presented complementary reviews of AI-driven wearable technologies for neonatal cardiorespiratory monitoring, with Part 1 focusing on wearable sensor types, designs, and modalities and Part 2 providing a hierarchical taxonomy of machine-learning (ML) and deep-learning (DL) methods for signal processing and classification. A narrative review by Magsayo and Khatami Firoozabadi (2025) [14] surveyed non-invasive pediatric wearables across cardiovascular, psychiatric, neurodevelopmental, and other applications, emphasizing design, privacy, and age-appropriate functionality but offering limited hospital-based validation. Together, these reviews offer valuable technical syntheses of sensor design, types, algorithmic approaches, and clinical contexts; however, an integrative perspective that bridges these overlapping domains—particularly linking wearable sensors, AI methods, and real-world hospital deployment—remains notably absent.

Our scoping review spans both hospital and hospital-supervised outpatient settings and covers the entire pediatric spectrum, from neonates to adolescents. We map device form factors, sensing modalities, and derived biosignals across diverse clinical domains, including oncology, surgery, and others, and evaluate how AI and analytical methods are integrated beyond basic physiological metric estimation toward outcome prediction, event detection, and clinical decision support. Furthermore, we analyze implementation barriers such as regulatory labeling, signal robustness, workflow integration, ethical and equity considerations, and pediatric-specific device design. By incorporating selected simulation-based usability and design-focused pediatric studies that provide translational insights, this review complements prior device- and algorithm-centric syntheses by offering a clinical and implementation-oriented perspective on the evolution of pediatric wearables and AI. The detailed research framework and specific study questions are presented in Section 2 (Methods).

## 2. Methods

### 2.1. Study Design

This study used a scoping review to map the breadth of pediatric wearables and AI use in hospitals and outpatient settings in the last decade and identify gaps and challenges regarding their increased adaptation.

Our research questions are broadly defined as follows:

1. What types of wearable biosensors are used in pediatric hospital and outpatient settings?
2. Which clinical domains have seen the use of wearable devices?
3. How and to what extent have pediatric wearable devices incorporated AI beyond basic physiological metric estimation (e.g., HR, HRV, RR) to support outcome prediction, event detection, or clinical decision support?
4. What barriers remain to their broader clinical adoption?

### 2.2. Search Strategy

This review was conducted as a narrative synthesis with structured screening, recognizing the heterogeneity of pediatric wearable studies. We followed the Preferred Reporting Items for Systematic Reviews and Meta-Analysis Extension for Scoping Reviews (PRISMA-ScR) guidelines [15]. The protocol was defined and registered (INPLASY2025100096) [16].

**Figure 1:**
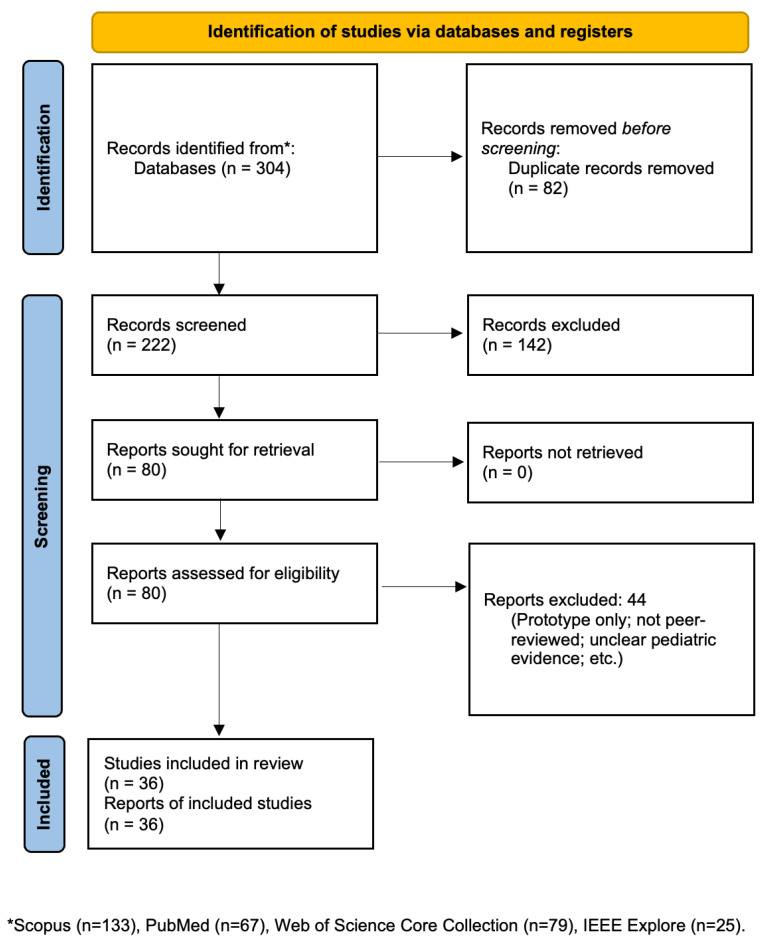
PRISMA flow diagram showing study selection for review.

A search of PubMed, Web of Science, IEEE Xplore, and Scopus was performed in August 2025, using combination of database-specific subject headings and text words. The search strings used for each database are presented in Supplementary Document S1. Manual screening of references in key review articles supplemented database results. The PRISMA flow diagram is presented in Figure 1.

### 2.3. Eligibility Criteria

We included original clinical studies reporting on the feasibility, validation, or outcomes of wearable devices used in hospital or outpatient pediatric populations. Studies published between January 2014 and August 2025 and available in English were included. A wearable device is defined as a sensor measuring various physiological signals, usually continuously and over brief-to-long durations. Additionally, the sensor should be noninvasive, i.e., does not penetrate the epidermis. Pediatric populations were defined as patients aged <18 years, though studies including participants up to 21 years of age were retained if conducted in pediatric clinical units or explicitly categorized as pediatric by the investigators. Both consumer-grade and medical-grade devices were eligible if deployed in real patients and capable of capturing physiological signals. AI algorithms broadly included ML and DL algorithms used for automated signal analysis.

The exclusion criteria were (1) adult-only studies (age ≥ 21 years), (2) mixed adult/pediatric cohorts without pediatric-specific reporting or unclear pediatric evidence, (3) simulation-only papers, (4) non-clinical prototypes, unclear pediatric evidence, and reviews, (5) conference abstracts without full peer-reviewed publications, (6) invasive, non-continuous, or one-time sensors, (7) sensor characterization without real pediatric patients, and (8) studies based in an at-home setting only, and (9) studies with insufficient methodological detail.

### 2.4. Data Extraction and Analysis

Data were extracted from the included studies and recorded in Microsoft Excel, including the year of the study, the title, the wearable sensor type and description, whether AI was used (and if so, the algorithm was included), the participant ages, and the disease/clinical domain. A second independent investigator reviewed the final studies for eligibility.

In this study, biosensors were classified based on their form factors, falling into categories such as multi-position band-type trackers, adhesive biosensor patches, textile-based systems, and special-purpose devices, including specific designs tailored for an application. These devices were associated with their clinical application domains.

## 3. Results

From an initial pool of 304 titles screened, 82 duplicates were removed, leaving 222 articles for title/abstract screening. Of these, 142 were excluded for not meeting the inclusion criteria. Eighty full texts were reviewed in detail, and forty-four were excluded. These included studies that mentioned pediatric participants but primarily analyzed adult data, combined pediatric and adult results without stratified reporting, or discussed pediatric applications only theoretically without presenting child-specific findings. Ultimately, 36 studies met the inclusion criteria. Each was abstracted into an evidence table capturing the year, title, device type, age range, disease focus, clinical setting, AI involvement, data modalities, whether the device was hospital-deployed, and outcomes of interest.

The deployment of WDs in pediatrics has centered on clinical domains where continuous, non-invasive monitoring offers the greatest potential benefit. Across oncology, surgery, cardiology, respiratory monitoring, and rehabilitation, among others, various sensors and their derived signals have been used for patient monitoring. An overview of all 36 included studies and their characteristics can be found in Table S1 (Supplementary Materials), which summarizes the device type, clinical focus, AI application, and main outcomes of these studies. The following sections describe these WD modalities and their clinical areas.

### 3.1. Device Types and Measures

**Figure 2:**
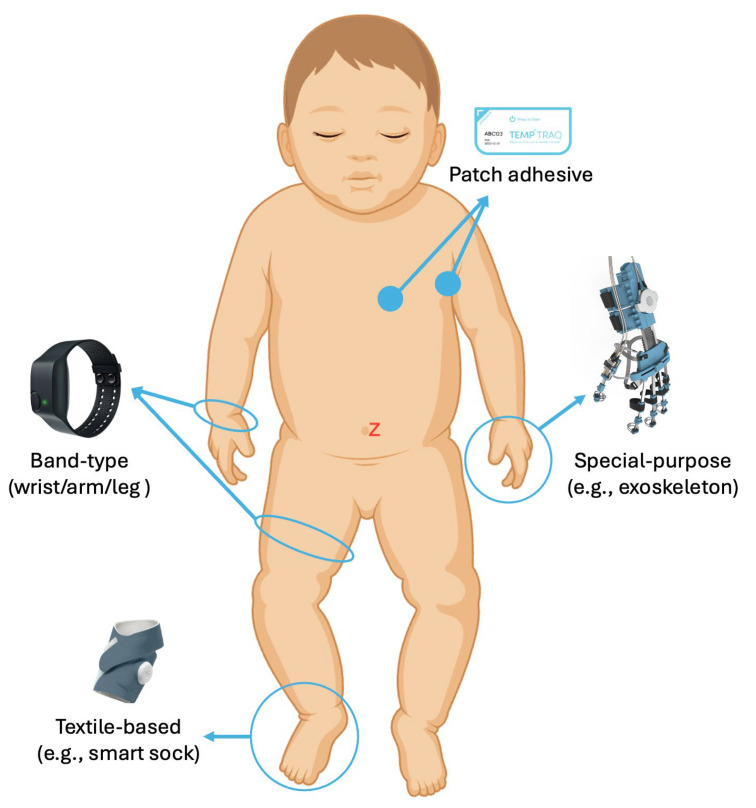
Illustration showing four overlapping categories of WDs in pediatrics: (1) band-type trackers, (2) adhesive biosensor patches, (3) textile-based systems, and (4) special-purpose devices.

Pediatric wearable form factors can be categorized into four overlapping groups: multi-position band-type trackers, adhesive biosensor patches, textile-based systems, and special-purpose devices, as illustrated in Figure 2. Each modality represents a careful balance between user comfort, practical usability, and signal quality. These features and the specific biosignals measured guide their use case.

Across all categories, the sensing modalities include ECG, optical measures of blood flow using PPG and near-infrared spectroscopy (NIRS), mechanical motion via inertial measurement units (IMUs) including an accelerometer and gyroscope, EDA, acoustic signals, temperature, and continuous noninvasive blood pressure (NIBP). Each stream provides a potential biomarker for physiology, disease progression, or recovery. Importantly, the richness of these datasets positions pediatric wearables as ideal candidates for AI–driven analytics.

**Figure 3:**
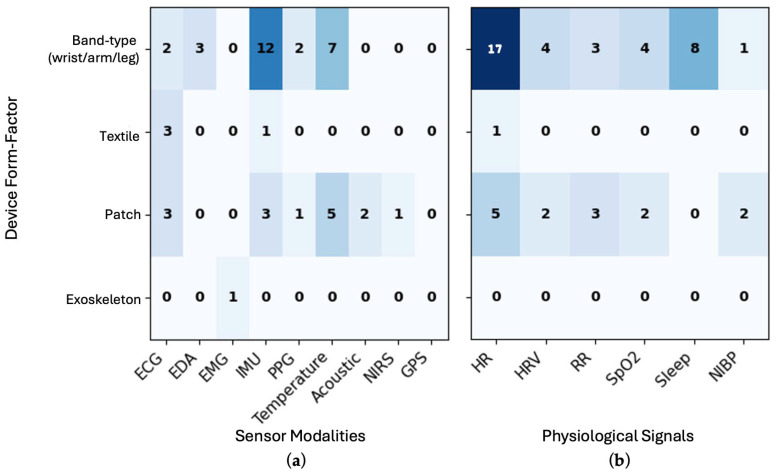
Heatmaps showing the association of four device form factors (band-type wrist/arm/leg worn, textile-based, adhesive patch and exoskeleton) with (**a**) sensor modalities and (**b**) derived physiological measures. Darker colors indicate a higher number of studies with given form-factor and sensor or physiological signal. Acronyms—ECG: electrocardiogram; EDA: electrodermal activity; EMG: electromyogram; IMU: inertial measurement unit; PPG: photoplethysmogram; NIRS: near-infrared spectroscopy; GPS: global positioning system; HR: heart rate; HRV: heart rate variability; RR: respiration rate; SpO_2_: oxygen saturation; NIBP: non-invasive blood pressure.

To provide a high-level overview of the research landscape, Figure 3 shows heatmaps of device form factors (band-type wrist/arm-worn, patch, textile-based, exoskeleton) and their associated sensor modalities (a) and derived biosignals (b) as evident from the literature. These heatmaps highlight a concentration of studies employing band-type and patch-based wearables, while textile and exoskeleton platforms are represented less frequently. Band-type devices most often integrate IMU (*n* = 12), temperature (*n* = 7), and PPG (*n* = 2) sensors. In contrast, patch systems display broader multimodal configurations, most commonly combining temperature and ECG sensing, with the occasional integration of IMU, PPG, or acoustic modalities for continuous physiological assessment. Derived biosignals were dominated by heart rate (HR) (Figure 3b). Band-type devices most frequently produced HR, heart rate variability (HRV), oxygen saturation (SpO_2_), and sleep-related metrics, consistent with their ambulatory use and integration of motion and optical sensing. In contrast, patch-based systems also captured HR, respiration rate (RR), SpO_2_, HRV, and occasional NIBP measurements. Together, these trends underscore a research focus on cardiorespiratory and thermal monitoring, with band-type devices optimized for activity and behavioral metrics, and patch systems supporting clinically oriented, high-fidelity physiological assessment.

Pediatric wearable devices employ diverse sensing modalities that translate physiological or mechanical signals into electrical outputs for continuous, non-invasive monitoring. Across the studies included in this review, four primary sensing categories were represented. Electrical sensors, including ECG and electromyography (EMG) electrodes, record biopotentials via Ag/AgCl or dry electrodes, providing a high temporal resolution (typically hundreds of hertz, e.g., 250–1000 Hz) and millivolt-level sensitivity suitable for detecting accurate HR and HRV [17,18]. Optoelectronic sensors, such as PPG and NIRS, measure changes in light absorption or scattering caused by pulsatile blood flow and oxygenation. These devices offer moderate sampling rates (25–200 Hz) and can achieve HR and SpO_2_ accuracy within 1–3% of clinical reference values, though performance degrades with motion [19]. Piezoelectric and piezoresistive sensors convert mechanical deformation into voltage or resistance changes for pressure, respiratory, or posture tracking. They typically operate at 10–200 Hz, display high strain sensitivity (gauge factors > 100), and have rapid response times (<10 ms), but require mechanical stabilization to minimize drift [20,21,22]. Thermistor-based sensors monitor cutaneous temperature variations with sub-degree precision and high stability over long wear periods, albeit with slower temporal dynamics [23]. Collectively, these sensing principles support the high-fidelity monitoring of cardiovascular, respiratory, and activity-related parameters in hospital and hospital-supervised outpatient environments. Other sensing domains, including electrochemical and microfluidic biosensors for biochemical analytes in sweat or interstitial fluid, are rapidly advancing in adult and preclinical research [24], but were not represented among the pediatric clinical studies identified here. These fundamental characteristics form the technical basis for the four wearable device form factors discussed below.

#### 3.1.1. Band-Type WDs

Wrist- and arm-worn devices are the most familiar form factors, adapted from both consumer technologies (e.g., Fitbit, Apple Watch) and medical-grade systems (e.g., Everion, Empatica). Their advantages are their ease of use, familiarity to families, and acceptability in outpatient and home settings. They commonly use PPG, IMU, and temperature sensors to derive HR, respiratory rate (RR), SpO_2_, physical activity estimates (e.g., step count), and skin and core temperatures. Advanced models incorporate single-lead ECG and can derive further outcomes such as sleep metrics, arrhythmia detection, and daily readiness score [25].

However, pediatric application is complicated by smaller wrist circumference, faster baseline HR and RR, greater susceptibility to motion artifacts, and developmental variability, all of which can limit the accuracy of PPG-derived signals, requiring different signal pre-processing steps [4,25,26]. Even with these challenges, these devices are increasingly being used in pediatric research, especially for monitoring during surgical recovery, epilepsy management, and outpatient cardiology [27,28,29].

#### 3.1.2. Patch WDs

Adhesive biosensor patches provide closer skin contact and multi-parameter monitoring in a single unit. Commercially available device such as VitalPatch use ECG, IMU, and thermistors to measure HR, HRV, RR, skin temperature, and core body activities with up to a 7-day battery life [30]. TempTraq is another similar device that measures continuous temperature with up to 72 h of battery [31], designed as a single-use disposable patch. Smart stethoscopes such as AeviceMD can be attached to the skin with a disposable silicon patch and enable automated acoustic analysis of wheezing and respiratory abnormalities [32].

Research-grade devices can provide more information with smaller form factors, such as the soft, skin-interfaced biosensors developed by Rogers’ research group [33,34,35]. They developed and tested a two-unit system [33]: a chest patch measuring ECG and skin temperature, together with a range of information extracted from a high-frequency three-axis accelerometer, such as seismocardiograms (SCGs) and respiration; and a limb unit measuring PPG, SpO_2_, and skin temperature. Together, these two units were used to estimate blood pressure (BP) using pulse transit time (PTT) [34]. From the same group, Rwei et al. described a soft, wireless, skin-interfaced sensor (33 × 16 × 3 mm; 2.8 g) with dual-wavelength LEDs (740/850 nm) and a four-photodiode array, enabling depth-resolved oximetry including both PPG and NIRS to derive cerebral oxygenation (ScO_2_), peripheral SpO_2_, HR, and surrogates of cerebral vascular tone [35]. Relative to traditional tethered NIRS, the advantages of this device include its cable-free operation, miniaturized/soft construction that reduces iatrogenic skin-injury risk, magnetically swappable batteries for uninterrupted wear, and simultaneous systemic and cerebral sensing, enabling scalable, real-time monitoring in both advanced and resource-limited settings. Tzavelis et al. described a miniaturized mechanoacoustic sensor (MAS) for pediatric cystic fibrosis (CF) monitoring that continuously measures coughing and vital signs across clinic, hospital, school, and home settings. The suprasternal-notch patch houses dual IMUs (*z*-axis sampled at 1.6 kHz) with onboard logging/BLE streaming and firmware compression, enabling > 24 h sessions and multi-day wear [36].

High-frequency data streams from these wireless patches have fewer motion artifacts, leading to higher quality data than that available from bedside monitors, while reducing the burden of repeated vital sign checks [37,38]. They are especially promising in oncology wards, intensive care, and sepsis monitoring. Yet, patches introduce challenges such as adhesive intolerance in neonates, skin irritation, and detachment during daily activity [39]. Compliance is often lower in younger children, underscoring the importance of pediatric-specific wearable design.

#### 3.1.3. Textile-Based WDs

Textile-based systems embed sensors into everyday garments such as shirts, chest bands, and full-body suits. Textile electrodes can capture ECG, and integrated accelerometers track movement and posture. By blending monitoring into everyday clothing, these platforms enhance comfort and reduce stigma, though they remain prone to motion artifacts and sensor dropouts. Early feasibility studies indicate acceptable accuracy alongside strong patient preference. For example, Nikolova et al. piloted a pediatric “intelligent textile suit” that continuously captured ECG and transmitted signals wirelessly, showing diagnostic-quality recordings in newborns to 10-year-olds without restricting mobility [40]. Similarly, Ghahjaverstan et al. evaluated a knitted smart textile chest band (SKIIN) in 20 children and adolescents, including those with congenital heart disease, and demonstrated ECG accuracy within ~3–4% of reference standards during rest and movement, with high acceptability among families [41]. Other emerging technologies like smart socks for infant vital signs fit within the broader class of apparel-based sensors that prioritize comfort and unobtrusiveness for continuous home monitoring [42]. These devices are designed to function as child-acceptable comfortable garments first and devices second, integrating seamlessly into routines and social contexts while adapting to growth and day-to-day variability to sustain adherence and therapeutic impact [43]. Thus, their future value may lie in rehabilitation, long-term outpatient monitoring, and seamless ward-based telemetry.

#### 3.1.4. Special-Purpose WDs

Purpose-specific special devices, generally therapeutic in nature, highlight the breadth of wearable innovation beyond passive monitoring. Pediatric exoskeletons provide rehabilitative support for neuromotor impairment [44]. Ritchie et al. proposed a holistic, lifestyle-centric approach to pediatric rehabilitation wearables for children with cerebral palsy [43]. Using a literature review, clinician interviews, ethnographic observations, and iterative prototyping, they identified three non-negotiable design imperatives—discretion, adjustability, and independence—and translated these into a soft-suit exoskeleton concept (“Myostep”) with modular components, growth-accommodating adjustment points, and wrap-style closures aimed at self-dressing and everyday usability. Wearable cardioverter-defibrillators, such as LifeVest, have been deployed in adolescents at risk of sudden cardiac death [45]. These illustrate how wearable technologies can be applied to active intervention and therapy.

### 3.2. Clinical Domains and AI Applications

**Figure 4:**
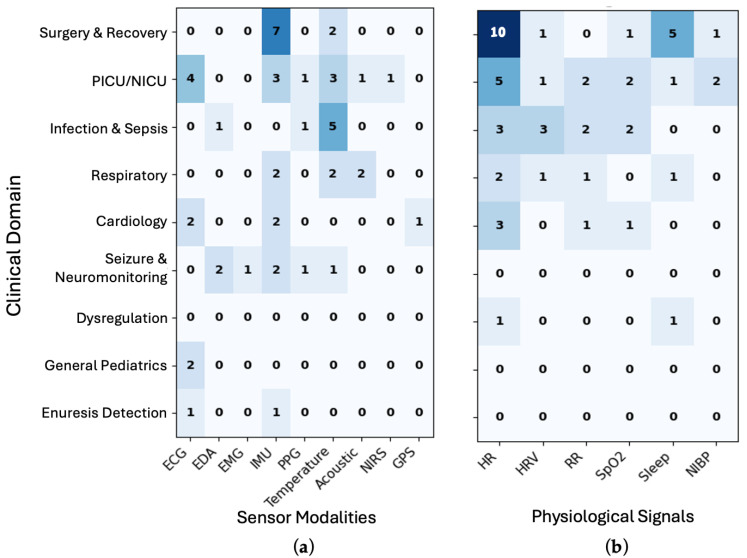
Heatmap showing association of clinical domains with (**a**) sensor modalities and (**b**) derived physiological measures. Darker colors indicate a higher number of studies with given clinical domain and sensor or physiological signal. Acronyms—ECG: electrocardiogram; EDA: electrodermal activity; EMG: electromyogram; IMU: inertial measurement unit; PPG: photoplethysmogram; NIRS: near-infrared spectroscopy; GPS: global positioning system; HR: heart rate; HRV: heart rate variability; RR: respiration rate; SpO_2_: oxygen saturation; NIBP: non-invasive blood pressure.

The application of WDs has progressed beyond feasibility testing toward preliminary diagnostic evidence and predictive use across diverse pediatric clinical domains, supported by a range of sensor modalities and physiological signals. To contextualize these findings, Figure 4a,b summarize the association between sensor types and derived biosignals across clinical domains identified in our corpus. As shown in Figure 4a, the use of ECG, temperature, and IMU sensors varies across clinical domains. Surgery and recovery studies relied heavily on IMU (*n* = 7) for mobility and recovery assessment, whereas PICU/NICU investigations frequently incorporated ECG (*n* = 4), IMU (*n* = 3) and temperature (*n* = 3) for multimodal cardiorespiratory monitoring. Infection and sepsis applications often utilized temperature (*n* = 5) to track inflammatory and febrile responses. Studies focusing on seizure and neuromonitoring leveraged EDA and IMU (*n* = 2), with a combination of EMG, temperature, and PPG (*n* = 1), to capture autonomic and motion-based signatures. In terms of derived signals (Figure 4b), HR was the most consistently measured parameter, particularly in surgery and recovery (*n* = 10), PICU/NICU (*n* = 5), and infection/sepsis (*n* = 3) settings. Overall, these findings highlight that cardiorespiratory sensing remains the foundation of pediatric wearable research, while applications in neurologic and behavioral domains are emerging.

Continuous and diverse high-frequency data streams from WDs have supported a spectrum of AI approaches: ridge regression for sepsis prediction [30], balanced random forests for appendectomy recovery [46,47], and convolutional neural networks (CNNs) for seizure and rhythm classification [48,49]. While results consistently demonstrate predictive utility, most models are trained on modest, single-center cohorts, and external validation across devices and populations remains rare. 

#### 3.2.1. Infection and Sepsis

Reliable continuous vitals monitoring is crucial for the management of pediatric patients receiving chemotherapy or recovering from surgical infections. WDs can be applied with high comfort to provide long-term vitals monitoring and provide predictive outcomes to escalate care.

In oncology wards, Everion devices have been tested across Swiss centers. An initial feasibility study at Inselspital Bern University Hospital followed 20 children aged 2–16 years undergoing chemotherapy, recording continuous multiparameter vital signs including HR, HRV, SpO_2_, RR, temperature, and skin blood perfusion. While families accepted the devices despite size and connection issues, good-quality HR was only available in 61% of hours due to compliance challenges [50,51]. Another study at the University Children’s Hospital Basel monitored 21 4–17-year-olds with perforated appendicitis, osteomyelitis, and septic arthritis. The Everion device reliably measured HR and SpO_2_, while skin temperature readings were influenced by ambient factors such as blanket coverage, leading to lower values compared to tympanic measurements. Nevertheless, feasibility and acceptance remained high [52]. TempTraq single-use temperature patches detected fevers 5–12 h earlier than oral thermometry in patients with febrile neutropenia, leading to timely antibiotic initiation in bloodstream infections [31]. Another multi-sensor comparison studies in two Swiss hospitals showed that while CORE devices provided accurate core temperature estimates (bias −0.07 °C vs. ear), Everion devices added HR, HRV, and RR, highlighting a trade-off between accuracy and signal breadth [53,54].

Perhaps the most striking example of wearables in oncology and infection surveillance comes from Dhaka Hospital ICU (Bangladesh), where VitalPatch biosensors were tested in 100 2-month to 17-year-olds admitted with sepsis. The devices achieved median > 99% high-quality data capture. Using VitalPatch data alone, the ridge regression model achieved an AUC of ~0.78 for predicting progression to advanced sepsis (pSOFA > 8). The model was trained on features derived from HR, RR, and skin and core temperatures, along with ECG and accelerometry. A combined model that also incorporated two manually measured variables improved performance (AUC 0.86) and detected deterioration up to two hours before clinician recognition. While these results highlight the feasibility of continuous wearable monitoring in low-resource settings, sepsis prediction remains a major challenge, and further validation in larger, multicenter cohorts is essential before clinical adoption [30].

#### 3.2.2. Surgery and Recovery

Surgical recovery is the area with the richest body of pediatric wearable evidence, particularly from an appendectomy recovery cohort. At the Ann & Robert H. Lurie Children’s Hospital (Chicago), 162 children aged 3–17 years wore a Fitbit device on their wrist for 21 days postoperatively, which passively tracked their activity, HR, and sleep, enabling recovery trajectories to be quantified. Analysis by De Boer et al. (2021) on an early small subset showed that patients with simple appendicitis reached a recovery plateau of ~8000 steps/day by postoperative day nine, while those with complicated appendicitis recovered more slowly and did not reach a plateau within the 21-day monitoring window [55]. In 2023, Ghomrawi et al. used balanced random forests to detect 83% of abnormal recovery days in complicated cases and 70% in simple appendicitis up to 48 h before symptom reporting [47]. A subsequent work in 2024 used an optimized balanced random forest framework to predict abnormal recovery three days before diagnosis at an accuracy of 87.5%, with improved pre-processing and labels [46]. In 2025, two articles demonstrated new metrics to track post-operative recovery trajectories. Hua et al. used novel circadian/ultradian biorhythm features to predict complications up to three days before diagnosis with 91% sensitivity and 74% specificity [56]. Pitt et al. validated pulse rate variability (PRVi) as a novel early biomarker—those with complications showed slower or declining PRVi before diagnosis [57]. Zeineddin et al. derived demographic-specific recovery curves for children after appendectomy using Fitbit step-count data, showing that age and BMI significantly affect recovery speed. This work demonstrated the feasibility of wearables for personalized discharge guidance [58].

Another work tested Fitbit HR both before and during elective pediatric surgery [59]. In this trial of 30 children aged 4–16 years undergoing laparoscopic or open procedures, Fitbit-derived values were collected every five minutes during anesthesia induction and intraoperative manipulation and compared with bedside monitors, demonstrating high accuracy for HR estimation compared to ECG, even in children under 8 years and <30 kg. Accuracy remained stable despite potential electromagnetic interference from surgical instruments.

Consumer wearable data have the potential to increase clinician confidence in remote patient management (RPM) by providing objective activity and HR trends that complement subjective symptom reports. In a multi-institutional simulation study across five U.S. children’s hospitals, Fitbit-derived HR and step count data meaningfully altered surgeons’ management of post-discharge telephone encounters, improving triage confidence and potentially reducing unnecessary ED visits [27].

#### 3.2.3. Seizure and Neuromonitoring

Epilepsy has driven the development of some of the most advanced wearable applications. A pivotal multi-center trial (Boston Children’s, NYU, Rhode Island Hospital, and others) tested the Empatica Embrace/E4 device in 85 pediatric patients aged 6–20 years. Using accelerometry and electrodermal activity, the device achieved seizure detection sensitivity of 0.92 in pediatrics with less than two false alarms/day, supporting FDA clearance [28]. Beyond this pivotal convulsive-seizure trial, a large study across epilepsy monitoring units at Boston Children’s and European centers evaluated wrist/ankle Empatica E4 devices in 166 patients (neonates–adolescents), with 900 recorded seizures. Using CNN and CNN–LSTM models based on accelerometry, EDA, blood volume pulse, and temperature data synchronized with video-EEG, the system detected 28 seizure types with 83.9% sensitivity. However, the false positive rate remained high at 35.3%, underscoring that while the approach extends utility well beyond tonic–clonic seizures, further refinement is needed before reliable clinical adoption can be achieved [48].

Another work evaluated the use of a digital health setup for seizure monitoring in a resource-limited setting in Cape Town, South Africa, where low-cost wrist WDs were paired with a mobile application. Caregivers reported 79% of seizure events digitally compared with just 5% via paper diaries, suggesting that RPM can be implemented, despite barriers like device cost, theft, and smartphone access [60].

Beyond seizure detection, pediatric neuromonitoring is expanding to continuous cerebral hemodynamics measurements. One clinical study validated a wearable patch with PPG and NIRS in an operating hospital environment across 12 pediatric subjects (0.2–15 y) with and without congenital central hypoventilation syndrome. Strong agreements were found (Bland–Altman bias ± SD: ScO_2_ 0.01 ± 0.9%; SpO_2_ 0.35 ± 0.63%; HR −0.2 ± 1.51 bpm), reproducing expected responses during head-up tilt, hyperoxia, hypoxia, and hypoxia–hypercapnia [35].

These findings illustrate how pediatric neurology has become a proving ground for wearable technology, ranging from FDA-cleared seizure detection to digital reporting tools, offering objective, continuous monitoring that complements caregiver observation and enhances clinical management.

#### 3.2.4. Respiratory Illness

Respiratory monitoring with consumer wearables is beginning to show clinical promise. In a study on 36 children with CF, the team collected > 2700 h of data and trained a ResNet-50 CNN on wavelet scalograms of accelerometer “burst” events to distinguish coughing from throat-clearing, speech/laughter, and motion artifacts, achieving an overall AUROC of 0.96 (AP 0.93). Longitudinal case series showed that MAS-derived cough counts, intensity, and physiologic measures (HR, RR, skin temperature, activity, and body orientation) tracked symptom surveys and clinical milestones (e.g., increased coughing during vest therapy and a decline before discharge). Overall, this work demonstrates a child-acceptable, AI-enabled pathway to objective, real-world cough surveillance and multiparameter physiologic monitoring in CF, with high usability and acceptance [36].

In a pediatric feasibility study at the National University Hospital, Singapore, AeviceMD demonstrated 85.3% sensitivity and 80.8% specificity for wheeze detection in the emergency department, with performance improving to >92% in home environments [32]. At Juliana Children’s Hospital (The Hague, Netherlands), Withings smartwatches were used to monitor 2–12-year-olds recovering from pneumonia, preschool wheezing, or asthma, capturing condition-specific recovery patterns: the median step-count recovery was 12 days for pneumonia, 5 days for wheezing, and 6 days for asthma. Step trajectories closely mirrored symptom scores, while daytime HR declined in wheezing and asthma but remained elevated in pneumonia, underscoring the ability of wearable devices to capture distinct physiological signatures during pediatric respiratory recovery [61].

#### 3.2.5. Cardiology

Cardiac monitoring has been another major use case for pediatric wearables. At St. Louis Children’s Hospital, Apple Watch Series 6 devices were evaluated in 84 patients ranging from infants to young adults, including many with congenital heart disease. SpO_2_ values differed by only 2.0 ± 2.6% from hospital-grade oximeters; manual interpretation of single-lead ECG intervals showed high agreement with 12-lead ECGs. However, the device’s automated rhythm classifier had only 75% specificity, with frequent inconclusive results in 23% of the cases. Younger patients posed difficulties in ECG measurements with the Apple Watch Series 6 due to a lack of coordination [29].

Beyond diagnostics, therapeutic devices have also been used. A U.S. multicenter registry of 455 children across 185 centers evaluated the ZOLL LifeVest wearable cardioverter-defibrillator, reporting 1.3% patients receiving appropriate therapy (100% survival of treated events), 0.4% receiving inappropriate shocks, and a median wear time of 20.6 h/day in high-risk adolescents [45].

#### 3.2.6. Rehabilitation 

Rehabilitation applications illustrate the diversity of pediatric wearables. At the University Children’s Hospital Zurich, the PEXO pediatric hand exoskeleton was piloted in a 6-year-old post-stroke child, demonstrating safe, task-oriented grasping training and potentially promoting neuroplasticity for increased rehabilitative effect [44]. An ongoing iTONE trial at the Children’s Hospital of Philadelphia is studying the impact of digitally supervised home-based rehabilitation program for children and adolescents (8–18 years) with pulmonary hypertension. The 16-week pilot combines ActiGraph smartwatches and Polar armband HR monitors with tailored exercise prescriptions, real-time monitoring, and two-way communication between patients and clinicians [62]. In the UK, consumer wearables paired with disease-specific smartphone apps were piloted across three hospitals in children with Niemann–Pick C, Duchenne muscular dystrophy, and juvenile idiopathic arthritis. The devices captured disease-specific ambulation differences, hence proving their utility for physical activity assessment in these diseases; however, complete data capture was not sustained [8].

An early clinic-integrated example comes from the Dyn@mo program at Sainte-Justine University Hospital Center, Montreal, Canada. Thirty-seven children aged 6–17 years with cardiometabolic risk wore a hip accelerometer (ActiGraph ActiTrainer activity monitor), a Polar Wearlink Heart transmitter chest monitor, and a handheld GPS for 7 days. The “spatio-behavioral” indicators were shown in an interactive application and reviewed together by clinicians and families to tailor goals and support individualized counseling rather than generic prescriptions. The study demonstrated how multi-sensor wearables can be operationalized inside pediatric care pathways for behavior change [63].

#### 3.2.7. Intensive Care

In critical care, soft, skin-interfaced biosensors have been tested extensively in neonatal and pediatric intensive care units (NICU/PICU). At Lurie Children’s Hospital and Northwestern Prentice Women’s Hospital (Chicago), Chung et al. demonstrated the feasibility of using wireless chest and limb biosensors in neonates and infants (premature 23 weeks gestational age to ~4 years) to capture HR, SpO_2_, and RR with high reliability and accuracy, in addition to advanced signals such as SCG, body orientation, activity, and vocal biomarkers [33]. A subsequent Lurie PICU study extended this work to BP monitoring using PTT calibration, correlating with invasive arterial lines (r = 0.85 for systolic BP, r = 0.77 for diastolic BP) across 23 critically ill children, thus demonstrating a noninvasive alternative to vascular cannulation [34].

#### 3.2.8. Enuresis

Expanding beyond postoperative monitoring, at-home nocturnal enuresis (NE) monitoring has also paired wearables with ML. Lee et al. describe NEcare, a pediatric system combining a belt-type bioimpedance and ECG device near the pelvis and bilateral ankle IMUs, coordinated using a phone gateway and cloud analytics [64]. Using data from 30 children during hospital urodynamics and 4 children monitored at home over 173 nights, they engineered a bioimpedance “delta” feature that tracked bladder filling better than raw signals (r ≈ 0.67 vs. 0.51). A convolutional–LSTM–attention model achieved up to 70.6% day-level NE moment estimation accuracy for one subject, demonstrating feasibility but underscoring the need for larger, externally validated cohorts and stronger generalization before clinical alarms can be automated.

#### 3.2.9. Psychiatric Dysregulation

Romanowicz et al. piloted smartwatch-derived biomarkers to anticipate disruptive behavior in ten 7–10-year-old patients hospitalized for the treatment of severe dysregulation [65]. Using a Garmin vivosmart 4 to passively collect minute-level HR, activity intensity, and sleep staging, they trained a supervised decision tree (DT) model to classify three states—calm, playful, and impending disruptive behavior within the next 60 min. Wearing adherence was high (~90%), and cross-validated accuracy reached 80.9% with a ~7.2% false-positive rate for the disruptive class. Feature analysis of DT showed that a HR > 129 bpm favored “playful” behavior, whereas a lower HR with extended light-sleep duration flagged a risk for imminent dysregulation. Although limited by a small sample size and an inpatient-only setting, this feasibility study shows that low-cost wearables plus simple ML can yield interpretable, real-time risk signals to trigger parent- or clinician-facing just-in-time support. 

### 3.3. Regulatory Landscape of Pediatric Wearables

In the United States, several wearable devices have received FDA clearance or authorization with explicit pediatric indications, although this list remains considerably shorter than that for adults.

Notable examples include physiologic monitors such as the Empatica Embrace wristband, cleared as an adjunctive detector of convulsive seizures for adults and children aged ≥6 years (K181861; prior adult-only clearance K172935) [66]. The TempTraq single-use temperature patch is indicated for individuals of all ages (K143267) [67]. Similarly, the Sibel Health ANNE Pediatric multi-parameter wireless patch is cleared for neonates (of any gestational age) to infants up to two years old (K221530) [68].

The ActiGraph LEAP holds 510(k) clearance (K231532) without specific age restrictions, providing continuous digital measures of activity, sleep, mobility, and physiological parameters including HR, HRV, and skin temperature [69]. The Owlet Dream Sock was granted de novo classification for infants aged 1–18 months to enable home pulse oximetry trend monitoring (DEN220091) [70].

Other cleared or authorized pediatric wearables include the ZOLL LifeVest wearable cardioverter-defibrillator (PMA P010030), indicated for children who meet defined size thresholds (e.g., ≥41 lb, chest ≥ 26 in) [71]. Most recently, the AeviceMD respiratory monitoring system received FDA 510(k) clearance (K223382) for pediatric use (ages 3 years and older), underscoring its translational potential as a regulated tool for remote respiratory assessment [72].

## 4. Practical Challenges

Despite the rapid expansion of pediatric wearable studies, several persistent challenges explain why most reports remain feasibility-focused rather than demonstrating hard outcomes or routine clinical adoption. These challenges span patient factors, device design, data quality, regulatory considerations, workflow integration, ethics, and equity.

### 4.1. Comfort, Fit, and Adherence

Children present unique ergonomic and developmental challenges. Consumer wrist devices designed for adults are often too big and sit loosely on smaller wrists/arm/legs [50], degrading PPG and IMU quality with increased motion artifacts. In an appendectomy recovery study, data gaps were common when devices were removed for bathing or discomfort [55]. Adhesive biosensor patches (e.g., VitalPatch) stabilize acquisition but are known to cause detachment with sweating and skin irritation, even in adults [73], and this has led to parental concerns being raised about fragile infant skin. In an oncology cohort, only 61% of hours yielded good-quality HR data, with no data during 35% of hours, underscoring the burden posed by adherence to the family [50]. Evidence from infant “smart socks” adds nuance: in a retrospective SVT cohort, home pulse-ox monitoring correlated with more phone and emergency-department encounters, yet also with better preserved left-ventricular function at presentation and a ~1.7-day shorter hospital stay, which suggesting that improved vigilance may increase alerts while enabling timelier care [74]. Taken together, these findings support the argument for for pediatric-first industrial design, including smaller housings and flexible bands, low-irritant adhesives, and improved battery life to minimize gaps and sustain high-quality data capture.

### 4.2. Signal Quality and Reliability 

Compared with bedside monitors, wearable signals remain variably reliable. Intraoperative Fitbit HR tracking in children was accurate but susceptible to motion/smaller wrists [59]. Apple Watch validation showed strong manual ECG interval agreement but frequent inconclusive automated rhythms in younger patients [29]. In intensive care, skin-interfaced biosensors matched invasive monitors for core vitals and even enabled NIBP estimation but still required careful placement and robust processing [33,34]. Motion artifacts, variable pediatric physiology, and limited data to train ML models all contribute to false alarms or missed detections, challenging the clinical reliability of wearables when used to trigger interventions. Collectively, this necessitates a specific validation of pediatric WD concepts rather than the “out-of-the-box” application of adult WDs.

### 4.3. Regulatory and Labeling Constraints

Device approval and labeling pathways further complicate translation. Many appendectomy dataset-based studies relied on a consumer tracker without pediatric medical indications, limiting formal adoption despite promising performance [46,47,55,57]. Medical-grade patches (e.g., VitalPatch) carry stronger credentials, but age-band labeling and algorithm approval constrain claims [30]. Once predictions influence care, algorithms become regulated functions, difficult to approve with small, heterogeneous pediatric datasets.

### 4.4. Workflow Integration and Alert Governance 

Most deployments stop short of EHR integration. Oncology projects often stored data on research servers, with offline data corrections [53]. Appendectomy programs similarly exported wearable data to offline pipelines; a rare multi-site simulation showed that clinicians changed referral decisions when given real wearable trends, but real-world wards still lack data standards, sync reliability, and alarm governance [27]. Finally, a recent survey study of nurses in Saudi Arabia [75] found generally positive attitudes towards AI-enabled wearables, but also emphasized a need for structured training, institutional support, and clear guidelines to facilitate optimal AI integration into clinical workflows.

### 4.5. Ethics and Equity Related Challenges 

Wearables generate sensitive, longitudinal traces of physiology and behavior. Continuous pediatric monitoring, often mediated by parents, raises questions about consent/assent, data minimization, and how (or whether) to return incidental findings. Field work in South Africa highlighted caregiver concerns around device theft, cost, and smartphone access, underscoring the need for transparent communication and pediatric-specific data-governance frameworks that clarify accountability and build family trust in how biosignals are stored, analyzed, and shared [30]. Equity barriers are amplified in low- and middle-income settings: even when high-quality data capture is achievable (e.g., >99% with medical-grade patches), success can hinge on reliable device supply, charging capacity, and cloud connectivity. Without designs robust to missingness, theft, and intermittent uploads, wearables risk widening disparities rather than narrowing them [30,60]. Methodologically, many AI models are trained on modest, often single-center datasets, limiting generalizability; label noise (e.g., defining “abnormal” recovery days or confirming true seizure events), firmware and device drift, and site-specific workflows further erode reproducibility over time [46,47]. Until externally validated across devices, ages, skin tones (affecting optical sensors), and care settings, pediatric models for postoperative complication detection and seizure monitoring should be interpreted cautiously and paired with multi-site training and continuous performance surveillance. 

### 4.6. AI Resource and Performance Trade-Offs

AI systems in healthcare need to carefully balance performance with practical constraints like time, cost and computational resources. Swaroop et al. (2024) [76] demonstrated that under time pressure, such as in emergency rooms, different AI-assistance strategies can impact the accuracy–time tradeoff and may not translate well from non-time-constrained environments. Additionally, implementing AI on edge devices for real-time monitoring introduces further trade-offs between limited computational resources and model accuracy [77]. Erion et al. (2022) [78] highlighted that conventional AI models optimize predictive accuracy without accounting for the downstream costs of deployment in clinical workflows, which can lead to economically unfeasible solutions. They demonstrated a cost-aware framework that substantially reduces the cost while maintaining strong performance. Furthermore, Alsahfi et al. (2025) [79] identified the infrastructural challenges of conventional cloud-based AI and proposed a regional computing paradigm to enable timely and efficient data access for clinical care. Collectively, these studies highlight both the challenges and recent advancements in addressing the performance-resource trade-offs inherent in healthcare AI deployment.

## 5. Future Directions and Conclusions

Pediatric wearables have progressed from novelty to credible clinical tools, but evidence still centers on their feasibility and validation. However, a growing number of studies have demonstrated how AI/ML can transform high-frequency biosignals into predictive tools, especially in postoperative recovery, sepsis detection, epilepsy monitoring, cardiac rhythm classification, and respiratory assessment. Collectively, these works show how wearable-derived data can move pediatrics from descriptive monitoring toward proactive, data-driven decision support.

The next steps include outcome-focused trials, multi-site datasets, and workflow-embedded systems. The priorities for research are as follows: (1) long-term chronic-care integration (e.g., rehabilitation and remote follow-up) [44,62]; (2) interoperable pipelines with EHRs and governed alerts; (3) multi-modal, explainable AI with external validation (e.g., seizure detection and iECG classification) [48,49]; and (4) equity-first designs robust to intermittent power/connectivity [30,60] and (5) recognizing and mitigating performance-resource trade-offs for clinical decision making with AI [76,78,79].

If these challenges are addressed, pediatric wearables can enable earlier detection, continuous surveillance, and individualized recovery trajectories, supporting proactive, personalized, and globally relevant pediatric care.

## Data Availability

No new data were generated in this study. The data supporting the findings of this review are derived from previously published studies, which are fully cited within the reference list.

## Supplementary Materials

The following supporting information can be downloaded at: https://www.mdpi.com/article/10.3390/bioengineering12121320/s1, Document S1: Database search strategies and screening criteria (PDF). Table S1: Evidence table of included studies (XLSX).
