# Supplementary material for: Integration of Artificial Intelligence and Wearable Devices in Pediatric Clinical Care: A Review
Source: Bioengineering (Basel). 2025 Dec 3;12(12):1320. doi: 10.3390/bioengineering12121320 (PMC12729400; doi:10.3390/bioengineering12121320)
Supplement: Supplementary file 1 [file bioengineering-12-01320-s001.zip › Database search strategies and screening criteria.pdf]

Databases: PubMed/MEDLINE, Web of Science Core Collection, IEEE Xplore, Scopus. Coverage window: January 1, 2014 to August 31, 2025. Languages: English at screening. Notes: Exact field tags and operators follow each database's conventions. Yields per database should be updated after running the queries.

A. PubMed (MEDLINE)
Search string (Boolean, with date filter in PubMed 'Advanced' builder): ((child*[tiab] OR pediatric*[tiab] OR paediatr*[tiab] OR "Child"[Mesh] OR "Infant"[Mesh] OR "Adolescent"[Mesh]) AND (wearable device*[tiab]) AND (hospital*[tiab] OR inpatient[tiab] OR ward*[tiab] OR ICU[tiab] OR "intensive care"[tiab] OR PICU[tiab] OR NICU[tiab] OR "emergency department"[tiab] OR ED[tiab] OR "operating room"[tiab] OR "Hospitals"[Mesh] OR "Inpatients"[Mesh] OR "Intensive Care Units"[Mesh] OR "Intensive Care Units, Pediatric"[Mesh] OR "Intensive Care Units, Neonatal"[Mesh] OR "Emergency Service, Hospital"[Mesh] OR "Operating Rooms"[Mesh] OR "Wards, Hospital"[Mesh])) AND ("2014/01/01"[Date - Publication] : "2025/08/31"[Date - Publication])
Yield: n = 67

B. Web of Science Core Collection
Timespan: 2014-2025; Indexes: SCI-EXPANDED, SSCI, A&HCI, CPCI-S, CPCI-SSH, ESCI
TS=((child* OR pediatric* OR paediatr*) AND ("wearable device*" OR (wearable NEAR/1 device*)) AND (hospital* OR inpatient OR ward* OR "hospital ward*" OR ICU OR "intensive care" OR PICU OR (pediatric NEAR/3 "intensive care") OR NICU OR (neonatal NEAR/3 "intensive care") OR ("emergency" NEAR/3 department) OR ("operating" NEAR/2 room) OR "operating theatre"))
Yield: n = 79

C. Scopus
TITLE-ABS-KEY((child* OR pediatric* OR paediatr*) AND ("wearable device*" OR (wearable W/1 device*)) AND (hospital* OR inpatient OR ward* OR ICU OR "intensive care" OR PICU OR NICU OR "emergency department" OR ("operating" W/2 room))) Publication Years: 2014-2025
Yield: n = 133

D. IEEE Xplore
Query: ((("All Metadata":"child*" OR "All Metadata":"pediatric*" OR "All Metadata":"paediatr*") AND ("All Metadata":"wearable device") AND ("All Metadata":"hospital" OR "All Metadata":"inpatient" OR "All Metadata":"ward" OR "All Metadata":"ICU" OR "All Metadata":"intensive care" OR "All Metadata":"PICU" OR "All Metadata":"NICU" OR "All Metadata":"emergency department" OR "All Metadata":"ED" OR "All Metadata":"operating room"))) Publication Years: 2014-2025
Yield: n = 25

E. De-duplication and counts Total records identified (all databases): 304
After deduplication: 222
Title/abstract excluded: 142

Full-text assessed: 80
Full-text excluded (reasons: insufficient pediatric data; prototype-only; not peer-reviewed): 43
Studies included: 37
